# Cataract Surgery Performed by High Frequency LDV Z8 Femtosecond Laser: Safety, Efficacy, and Its Physical Properties

**DOI:** 10.3390/s17061429

**Published:** 2017-06-18

**Authors:** Bojan Pajic, Zeljka Cvejic, Brigitte Pajic-Eggspuehler

**Affiliations:** 1 Eye Clinic Orasis, Swiss Eye Research Foundation, 5734 Reinach AG, Switzerland; brigitte.pajic@orasis.ch; 2 Department of Physics, Faculty of Sciences, University of Novi Sad, Trg Dositeja Obradovica 4, 21000 Novi Sad, Serbia; zeljkac@uns.ac.rs; 3 Division of Ophthalmology, Department of Clinical Neurosciences, Geneva University Hospitals, Rue Gabrielle-Perret-Gentil 4, 1205 Genève, Switzerland; 4 Faculty of Medicine of the Military Medical academy, University of Defense, 11000 Belgrade, Serbia

**Keywords:** femtosecond laser, physical properties, cataract surgery, clinical outcomes, complications

## Abstract

Background: The aim of our study was to investigate the safety and efficacy of the LDV Z8 femtosecond laser in cataract surgery compared to the conventional procedure. Methods: This prospective study was performed at the Swiss Eye Research Foundation, Eye Clinic ORASIS, Reinach, Switzerland. The study included 130 eyes from 130 patients: 68 treated with femtosecond laser-assisted cataract surgery (FLACS) using the FEMTO LDV Z8 and 62 treated with conventional phacoemulsification. Capsulotomy and lens fragmentation in the laser group were performed with the FEMTO LDV Z8 femtosecond laser system, which employs a new, low-energy, high repetition rate laser process for cataract surgery. In the conventional group, the capsulotomy was performed by a cystotome, and lens fragmentation was achieved by the stop-and-chop. Results: Ease of phacoemulsification (on a 4-point scale), the completeness of capsulotomy (on a 10-point scale), effective phacoemulsification time (seconds), uncorrected distance visual acuity (UCVA), best spectacle-corrected distance visual acuity (BSCVA), spherical equivalent (SE), and safety of the procedure were evaluated. The total follow-up time was three months. Conclusions: FLACS with the FEMTO LDV Z8 system was characterized by complete and reproducible capsulotomy and highly effective lens fragmentation. Postoperative visual outcomes were excellent, and the safety of the procedure was optimal.

## 1. Introduction

Femtosecond laser technology was first introduced in corneal refractive surgery for performing LASIK flaps. Compared to the microkeratome, an increase in precision and safety was observed [1,2]. Femtosecond technology was soon expanded to cataract surgery where its application introduced a higher predictability, safety, and potentially improved refractive outcome. Femtosecond laser systems are designed to perform anterior capsulotomy, lens fragmentation using different patterns, clear corneal incisions, and arcuate incisions. In the past few years, a significant amount of research investigating the advantages of femtosecond laser-assisted cataract surgery compared to conventional ultrasound phacoemulsification cataract surgery has been conducted. These studies have demonstrated that femtosecond laser-assisted capsulotomies have a higher precision regarding circularity and placement versus achieved diameter than those created by manual continuous curvilinear capsulorhexis. The femtosecond laser lens fragmentation results in a significant decrease in phacoemulsification energy exposure to the eye [3,4,5,6,7,8]. In the literature, it has been shown that the cut quality of clear corneal incisions done by femtosecond laser improves the tunnel morphology with less tissue damage compared with the conventional procedure. Due to its high precision and excellent surgical planning, femtosecond use in cataract surgery has also reduced the occurrence of high-order aberrations [9,10,11]. Femtosecond laser technology is, at the moment, more expensive compared to conventional cataract surgery but offers several advantages. A slightly greater amount of time is necessary for the femtosecond laser procedure compared with conventional phacoemulsification.

### Femtosecond Laser

**Figure 1:**
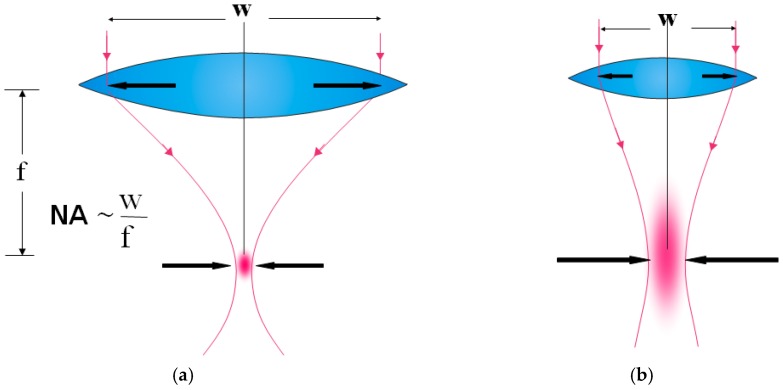
(**a**) The large numerical aperture is the key for minimizing the focal volume which leads to low pulse energy and less bubbles (LDV Z8 femtosecond laser). (**b**) The small numerical aperture leads to higher pulse energy and more bubbles (conventional laser systems).

The Femto LDV Z8 (Ziemer Ophthalmic Systems AG, Port, Switzerland) is a high frequency femtosecond laser system for corneal surgery, corneal-refractive surgery, and cataract surgery. Unique among cataract laser systems, the Z8 applies the concept of overlapping low-energy near-infrared (1030 nm) femtosecond laser pulses in the nano-Joule range. This concept was originally developed by Ziemer Ophthalmic Systems for corneal surgery but was later adapted for cataract surgery applications such as lens fragmentation, capsulotomy, clear corneal incisions, and arcuate incisions. In order to shorten surgery time because small spots with large numerical aperture are used (Figure 1a), the laser system runs in the MHz range applying up to 1 billion pulses per surgery. Conventional femtosecond lasers with small numerical apertures (Figure 1b) that run in the kHz range, leads to a larger spot with a higher pulse energy and more gas creation.

**Figure 2:**
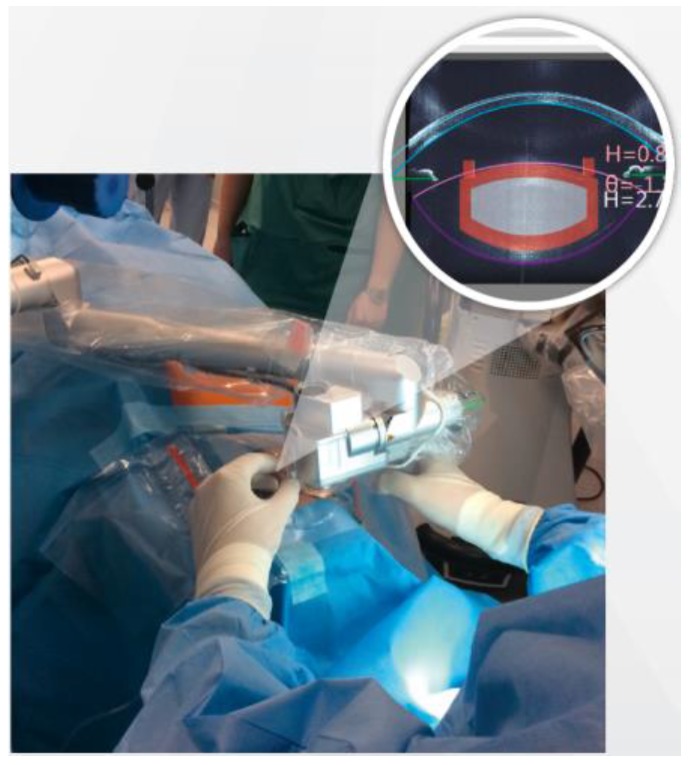
Femtosecond laser handpiece of the LDV Z8.

**Figure 3:**
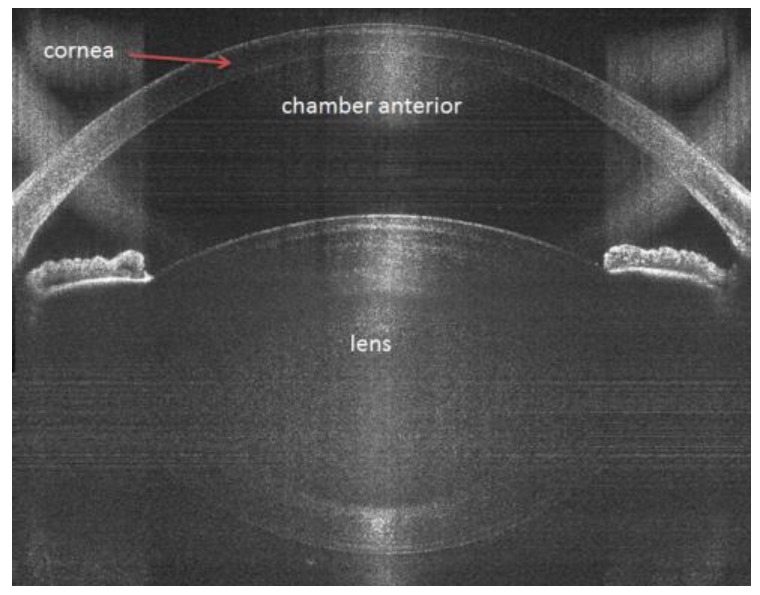
High-resolution OCT at a wavelength of 840 nm is mandatory for receiving a precise cut in the right position.

Each pulse creates a cavitation bubble approximately a few microns wide, which gently separates tissue. The handpiece (Figure 2) is the size of a compact camera and integrates all required electronics, optics, and actuators to perform visualization and resection in the anterior chamber of the eye. Visual resolution is possible down to 5 microns and is performed with a combination of a color camera and spectral-domain optical coherence tomography (OCT) operated at 840 nm (Figure 3).

**Figure 4:**
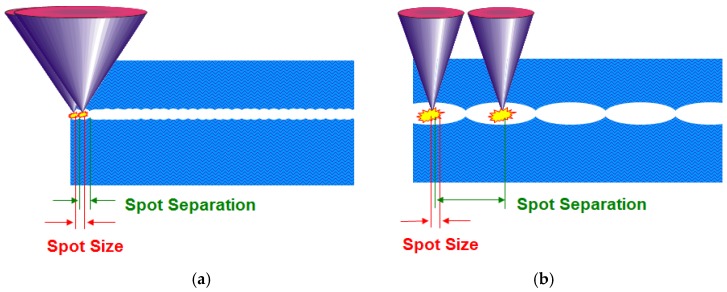
(**a**) The cutting process is limited to the focal spot size. Many pulses are needed to cut the tissue, so a high frequency repetition rate is needed. (**b**) The cutting process is mainly performed by mechanical forces of the expanding gas bubbles). Fewer pluses are needed but stress is generated in the tissue.

The FEMTO LDV Z8 uses a high focusing power microscope lens integrated in the handpiece to achieve focusing to a small spot size (<2 μm), which enables cuts to be made with nJ pulse energy. Low pulse energy with high-frequency applications is very precise and allows a spot diameter of less than 2 μm (Figure 4a). Conventional femtosecond lasers with a small numerical aperture and pulse energies in the µJ range has spot diameters greater than 5 μm and spot separation greater than the spot diameter (typically 10–20 μm), which can lead to tissue bridges (Figure 4b).

Given this unique combination of technical parameters, FLACS is able to create a more precise and circular capsulorhexis that could improve phacoemulsification and IOL centration, so a more precise refractive outcome after surgery could be expected [4,11,12,13,14,15].

## 2. Materials and Methods

This prospective randomized operative-interventional case–control study compares the performance and safety of femtosecond laser-assisted cataract procedures with those of conventional phacoemulsification cataract surgery.

The inclusion criteria required eligibility to undergo lens extraction by phacoemulsification followed by IOL implantation, an ability to complete patient interface docking with the femtosecond laser, an age of 50 years of older, willingness and ability to return for scheduled follow-up examination, and no current infections. Exclusion criteria consisted of minimal and maximal K-values of the central 3 mm zone that differ by more than 5D on topographic map of the cornea, a maximum K-value that exceeds 50D, a minimum K-value of less than 37D, corneal disease or pathology, such as corneal scaring or opacity, that precludes the transmission of laser wavelength or that distorts laser light, poorly dilating pupils of less than 6 mm or any other defect of the pupil that prevents the iris from adequate refraction peripherally, manifest glaucoma and ocular hypertension, and pseudoexfoliation. Additionally, any systemic or ocular pathology or previous ocular surgery was also excluded. All surgery procedures were performed by an experienced surgeon (BP) at the Eye Clinic ORASIS between January 2015 and September 2016.

The Z8 is a mobile femtosecond laser system that can be used in a sterile environment of the operating theatre. The hand-held patient interface allows for surgery to be performed without making significant alterations to the operation room layout in terms of space and equipment, thus preserving existing workflows. In the current study, all femtosecond laser cataract surgeries and conventional cataract surgeries were performed under the surgeon’s microscope, and no patients were moved into or out of the operating room during the procedure. All surgeries were performed under topical anesthesia rapidocain intracameral. Preoperative the pupil dilation was achieved by application of Mydriasert (combination of phenylephrine hydrochlorid (5.4 mg) and Tropicamide (0.28 mg)).

### 2.1. Femtosecond Laser-Assisted Cataract Surgery Technique

**Figure 5:**
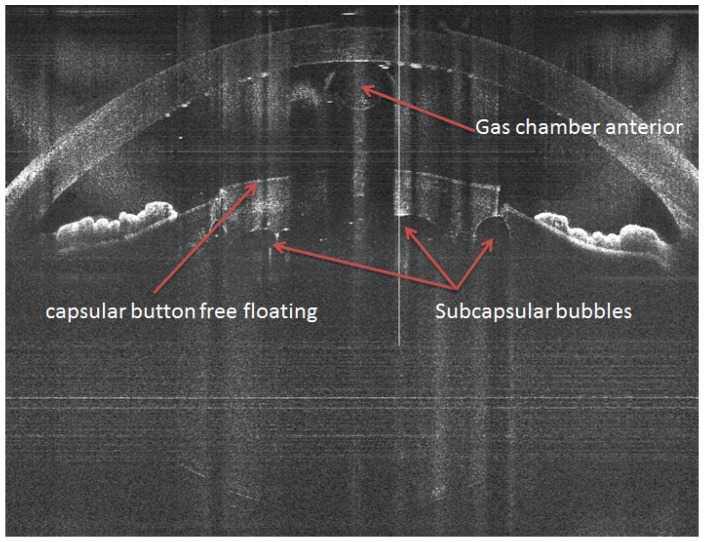
A complete capsulotomy is seen with the capsular button free floating in the anterior chamber, while there is nearly no accumulation of gas in the fragmented lens. There are minimal subcapsular bubbles in the anterior chamber.

The suction ring of a disposable liquid–patient interface was applied to the eye with centration over the limbus. The system contains a liquid interface (no applanation), which prevents posterior corneal descemet folds, ensuring an unhindered laser beam transmission. As soon as the suction vacuum reached 400 mbar, the suction ring was filled with a balanced salt solution (BSS). The handpiece, which is attached to an articulating arm of the laser system, was docked over the corneal apex. In the handpiece, there is a color camera and an integrated ocular coherence tomography (OCT) system that images the ocular structures. Treatment parameters were customized to accommodate each individual patient. Custom surgical planning is performed by the precise placement of surgical incisions based on OCT images that identify ocular structures and automatically determine and display safety margins and suggested cut locations. Via a touchscreen, the surgeon has the ability to reposition treatment patterns. Laser treatment begins with lens fragmentation (an eight-piece pie-cut pattern) followed by anterior capsulotomy (5.0 mm in diameter). Unlike other FLACS systems, lens fragmentation before anterior capsulotomy is possible with the Z8, as the low energy results in minimal gas production, which significantly reduces the risk of intra-operative complications. As shown in Figure 5, the capsular button in the OCT image can be seen free-floating, while the fragmented lens shows no accumulation of gas.

### 2.2. Conventional Cataract Surgery Technique

Two paracentesis 0.8 mm in diameter were performed. Intracameral anesthesia was administered (Lidocaine hydrochlorid and Epinephrine, 0.005 mg). A capsulorhexis 5 mm in diameter was attempted. For phacoemulsification, the stop-and-chop technique was applied. The remainder of the surgical steps required to complete the operation were identical between FLACS and conventional groups.

Minimal gas accumulation was visible at the posterior surface of the cornea, which was observed in the OCT through the creation of shadows seen on the lower part of the image. For all patients, the phacoemulsification device Catharex 3 system (Oertli Instrumente AG, Berneck, Switzerland) was used. By using the bimanual irrigation/aspiration system, residual cortex was removed. In all cases, an IOL was implanted in the capsular bag. Patients were scheduled for postoperative examination at Day 1, Day 12, and 4, 8, and 12 weeks after surgery. The main outcome measures evaluated in this study were best corrected visual acuity (BCVA), effective phacoemulsification time (EPT, seconds), and complications. Cataract severity was graded according to nuclear opalescence on the Lens Opacities Classification System III [16] with Grades 1–4.

Statistical analysis was computed with IBM SPSS Statistics version 20.0 (IBM Corp., Armonk, NY, USA). Normal distribution of data was determined by the Shapiro–Wilk test (data was considered normal if *p* > 0.05). Normal values were shown as mean ± standard deviation, whereas the median value was shown as non-parametric data. The level of significance was set at *p* < 0.05. BCVA was measured with Snellen projector charts, and data were converted to logarithm of the minimum angle of resolution (logMAR) units for statistical analysis. Related-samples Wilcoxon signed rank test was used to compare preoperative and postoperative BCVA and intended versus measured capsulotomy diameters.

## 3. Results

There were 33 males (48.5%) and 35 females (51.5%) in the FLACS group and 25 (40.3%) males and 37 females (59.7%) in the conventional cataract surgery group enrolled in the study.

The ethics committee of Northwest and Central Switzerland (EKNZ) approved the study. A total of 130 eyes from 130 patients were recruited for cataract treatment, with 68 patients recruited to the FLACS group (Group 1) and 62 patients recruited to the conventional cataract surgery group (Group 2). The mean age of patients in the FLACS group was 70.4 ± 8.4 years (range: 50–83 years) and 69.6 ± 8.2 years (range: 50–85 years) for the conventional cataract surgery group. There was no statistically significant difference in age (*p* = 0.25) between the two groups. Regarding the preoperative BCVA, there were no significant differences between the groups (*p* = 0.49). According to the Lens Opacities Classification System III, the preoperative cataract grade density in the femtosecond laser cataract surgery group was 2.57 ± 0.58 and the conventional cataract surgery group was 2.23 ± 0.42, which is highly significant (*p* < 0.001).

All patients in both groups underwent a successful operation. The intended capsulotomy diameter was set at 5.0 mm in all cases. The achieved capsulotomy diameter in the FLACS group was 5.0 ± 0.12 mm (range: 4.6–5.4 mm), median 5.0 mm, and in the conventional cataract surgery group 4.7 ± 0.36 mm (range: 4.0–5.6 mm), median 4.7 mm, which is highly significant (*p* < 0.001). The mean phacoemulsification time in Group 1 was 1.9 ± 2.25 s (range: 0–11 s), median 1.0 s, and in Group 2, 2.3 ± 2.41 s (range: 0.3–14 s), median 1.7 s, which is significant (*p* = 0.042). The effective phacoemulsification time (EPT) in Group 1 was found to be 1.48 ± 1.80 s (range: 0–8.8 s), median 0.8 s, and in Group 2 1.81 ± 1.93 s (range: 0.24–11.2 s), median 1.36 s, which is significant (*p* = 0.044) (Table 1).

Overall surgery time in Group 1 was 7.5 ± 1.22 min (range: 5–12 min) and 6.6 ± 1.76 min (range: 4.6–12 min) in Group 2, that is slightly significant (*p* = 0.048).

The mean preoperative BCVA was 0.29 logMAR (range 1.30–0.01 logMAR) in Group 1 and 0.30 logMAR (range 1.30–0.01 logMAR) in Group 2 (*p* = 0.50). There is no difference between the groups. The mean BCVA, 1 day post-operation, was 0.16 logMAR (range 1.30–0 logMAR) in Group 1 and 0.22 logMAR (range 1.30–0 logMAR) (*p* = 0.038) in Group 2; 12 days post-operation, 0.06 logMAR (range 0.49–0 logMAR) in Group 1 and 0.06 logMAR (range 0.60–(−0.10) logMAR) in Group 2 (*p* = 0.48); 4 weeks post-operation, 0.03 logMAR (range 0.49–(−0.10) logMAR) in Group 1 and 0.06 logMAR (range 0.30–(−0.10) logMAR) in Group 2 (*p* = 0.31); 8 weeks post-operation, 0.03 logMAR (range 0.40–(−0.10) logMAR) in Group 1 and 0.06 logMAR (range 0.49–(−0.10) logMAR) in Group 2 (*p* = 0.41); 12 weeks post-operation, 0.01 logMAR (range 0.30–(−0.10) logMAR) in Group 1 and 0.02 logMAR (range 0.49–(−0.10) logMAR) in Group 2 (*p* = 0.37). Only the value at 1 day is significant, whereas at any later follow-up there are no significant differences between the groups (Table 2).

The vacuum time for FLACS patients 139 ± 26 s.

Follow-up was 3 months for all patients. No intraoperative complications were recorded.

## 4. Discussion

Numerous studies have reported advantages of femtosecond laser over conventional phacoemulsification cataract surgery [3,4,6,8,13,14,17,18,19,20,21,22,23]. Our study shows that a targeted 5 mm capsulotomy could be achieved very precisely with the femtosecond laser treatment, demonstrating only small variance in reproducibility, whereas the manual capsulorhexis differed significantly. Other studies have demonstrated very similar results, where the capsulotomies created using the femtosecond laser were more accurate in size than those created by manual continuous curvilinear capsulorhexis [4,8,13,14,22]. The accuracy of the capsulotomy is very important because it provides the surgeon access to the capsular bag for fragmentation and removal of natural lens and placement of the IOL. The diameter of the capsulotomy should allow overlap between the capsul rim and the IOL optic and haptic for correct IOL positioning. In special IOL, like toric IOL, the alignment is particularly important. Thus, the diameter, shape, and centration of the capsulorhexis can influence IOL position and may have an impact on refractive outcomes.

According of Lens Opacities Classification System III, the preoperative cataract grade density in the femtosecond laser-assisted cataract surgery group (Group 1) was significantly higher (*p* < 0.001) than in the conventional cataract surgery group (Group 2) in our study. Despite this potential disadvantage, we needed significantly less phacoemulsification time and effective phacoemulsification time in the femtosecond laser group (Group 1) compared with the conventional phacoemulsification surgery group (Group 2). Other studies have shown that the reduction of ultrasound energy from phacoemulsification can reduce the risk of capsule complication. Phacoemulsification time and effective phacoemulsification time are known to increase with nuclear density, but other groups have reported similar findings regarding FLACS and EPT [4] as we have observed. In our study, it was seen that, even with a low-energy laser application, the lens fragmentation was perfectly cut. Low energy creates smaller gas bubbles, which reduces the tension on the capsular bag during the procedure. Thus, the Femto LDV Z8 femtosecond laser is able to perform the optimal procedure algorithm, where the cut procedure begins with the lens fragmentation followed by capsulotomy and clear corneal incisions. We did not observe any bubbles during any of the stages of the laser procedure, which could disturb the optimal cut quality.

In our study, we detected an improvement in visual acuity one day post-operation, an improvement that was significantly greater with the femtosecond laser compared with the conventional procedure. This may reveal that the rehabilitation time in the first postoperative day is faster. In all other follow-up times after 12 days, no significant differences regarding the visual acuity were observed.

FLACS with the LDV Z8, a low-energy high frequency femtosecond laser, shows very high precision, with a significant decrease in effective phacoemulsification time (EPT) compared to the conventional procedure, even when the lens density was higher. Our results indicate that the healing time involved in visual acuity is faster in the first postoperative days, demonstrating the clinical advantages of a gentle technique with the femtosecond laser. However, regarding the overall surgery time, we report that slightly more time is needed to perform femtosecond laser treatment, but the potential to optimize workflow further and eliminate this difference is a future aim.

## 5. Conclusions

One of the main goals is providing repeatable and precise outcomes, with an aim to offer customized medical solutions for patients. The low-energy LDV Z8 with its advanced OCT visualization allows the best placement of the capsulotomy and optimizes surgical planning. All of this influences the strength of capsulotomy and significantly improves refractive outcomes due to reduced IOL tilt [14,24]. A high-frequency device with low pulse energy enables minimal gas creation during lens fragmentation. This sets it apart from other cataract laser devices with higher energy and low frequency, where larger bubbles and tissue bridges are created.

## Figures and Tables

**Table 1:** Mean phacoemulsification time/effective phacoemulsification time for Groups 1 and 2.

	Group 1	Group 2	*p*-Value
mean phacoemulsification time (s)	1.9 ± 2.25	2.3 ± 2.41	0.042
effective phacoemulsification time (EPT)	1.48 ± 1.80	1.81 ± 1.93	0.044

**Table 2:** Mean BCA for Group 1 and Group 2 in the follow-up.

Mean BCVA	Group1	Group2	
preoperative	0.29 logMAR (range 1.30–0.01)	0.30 logMAR (range 1.30–0.01)	*p* = 0.50
1 day post-operation	0.16 logMAR (range 1.30–0)	0.22 logMAR (range 1.30–0)	*p* = 0.038
12 days post-operation	0.06 logMAR (range 0.49–0)	0.06 logMAR (range 0.60–(−0.10))	*p* = 0.48
4 weeks post-operation	0.03 logMAR (range 0.49–(−0.10))	0.06 logMAR (range 0.30–(−0.10))	*p* = 0.31
8 weeks post-operation	0.03 logMAR (range 0.40–(–0.10))	0.06 logMAR (range 0.49–(−0.10))	*p* = 0.41
12 weeks post-operation	0.01 logMAR (range 0.30–(−0.10))	0.02 logMAR (range 0.49–(−0.10))	*p* = 0.37
